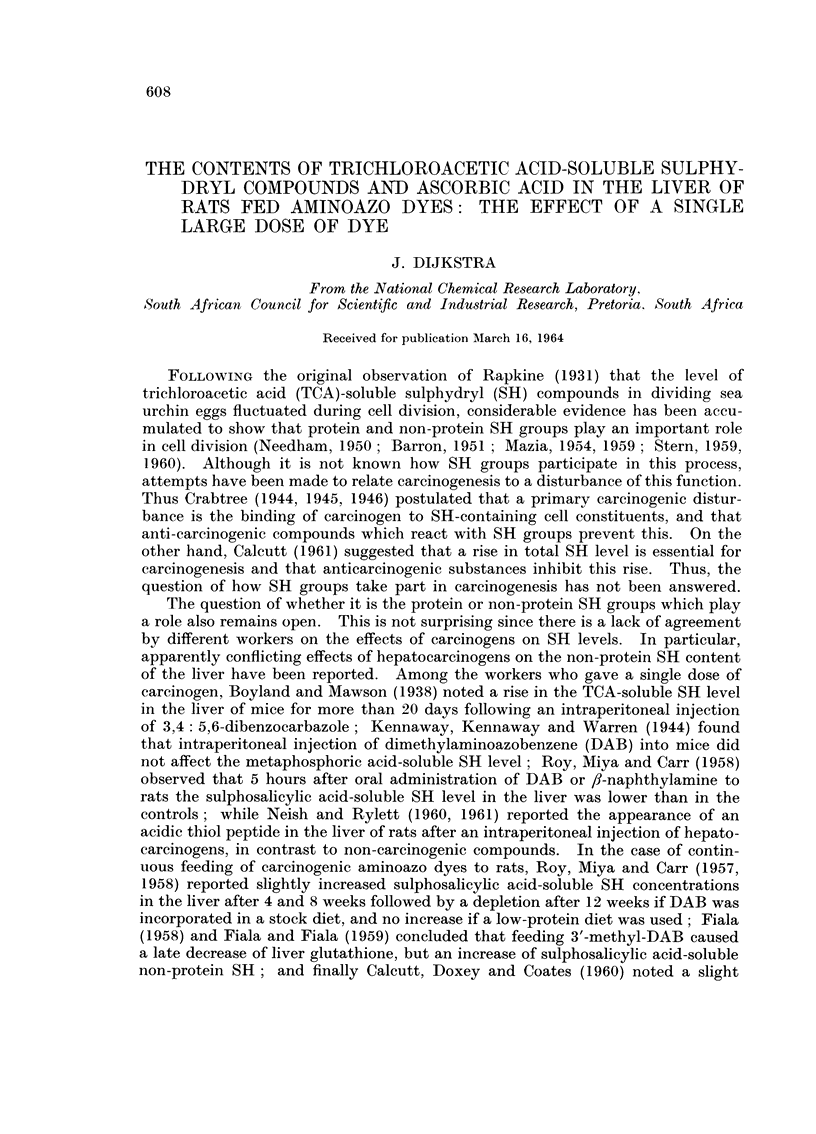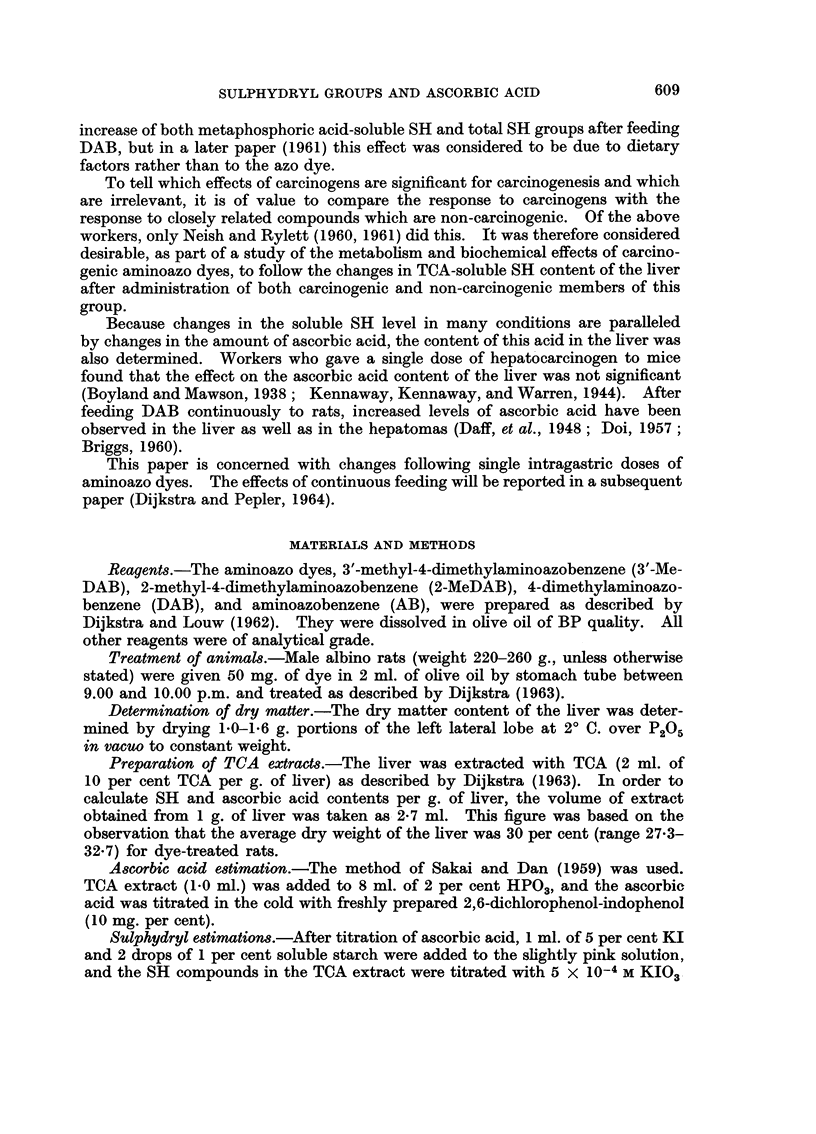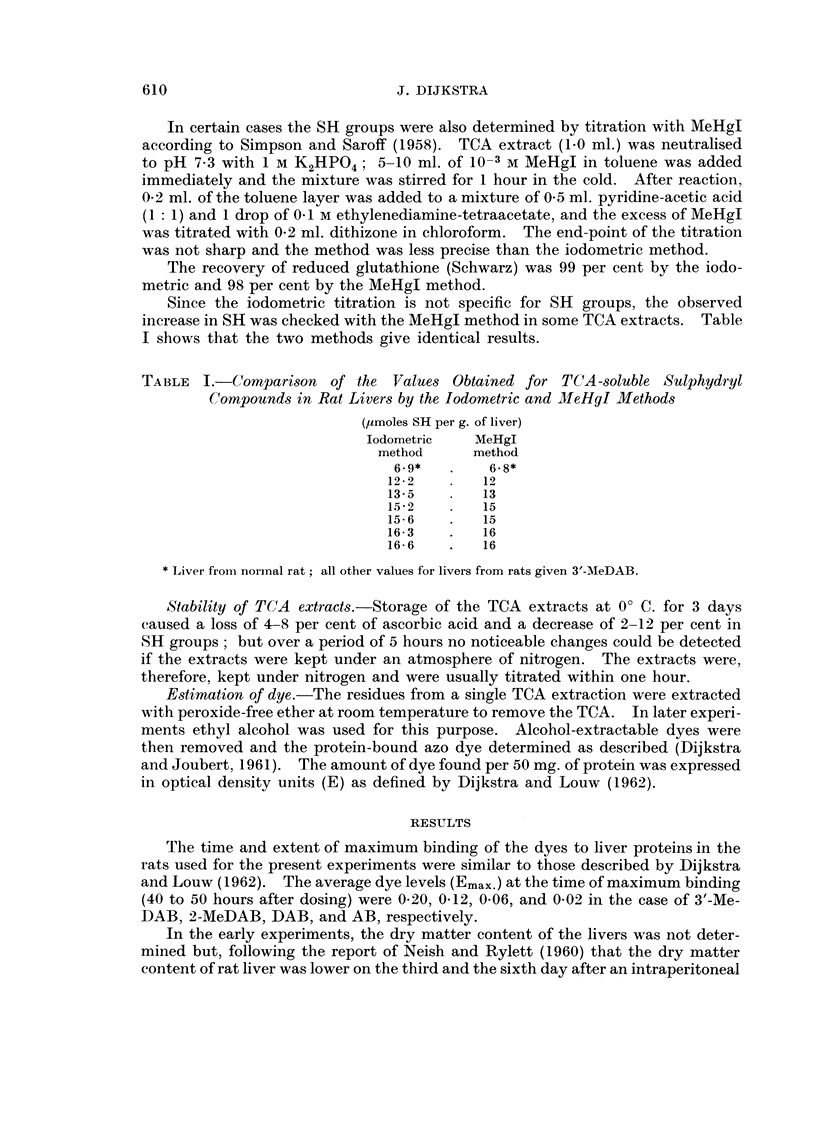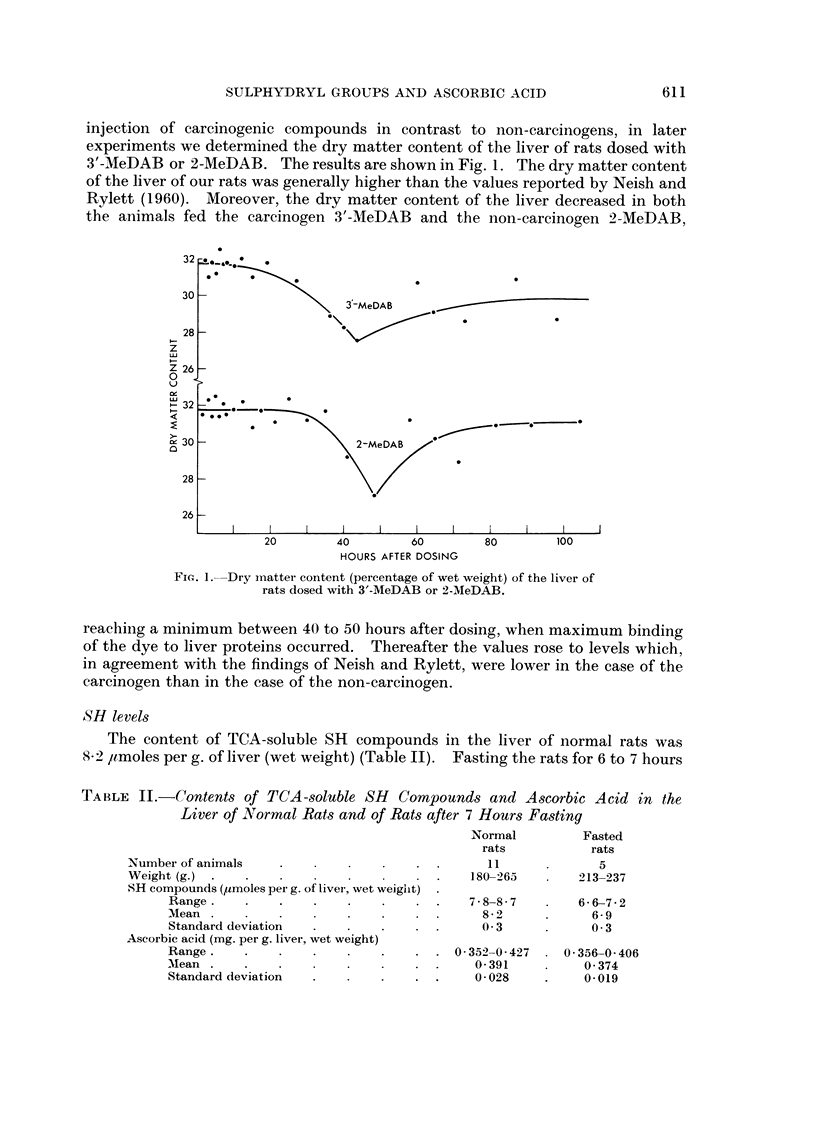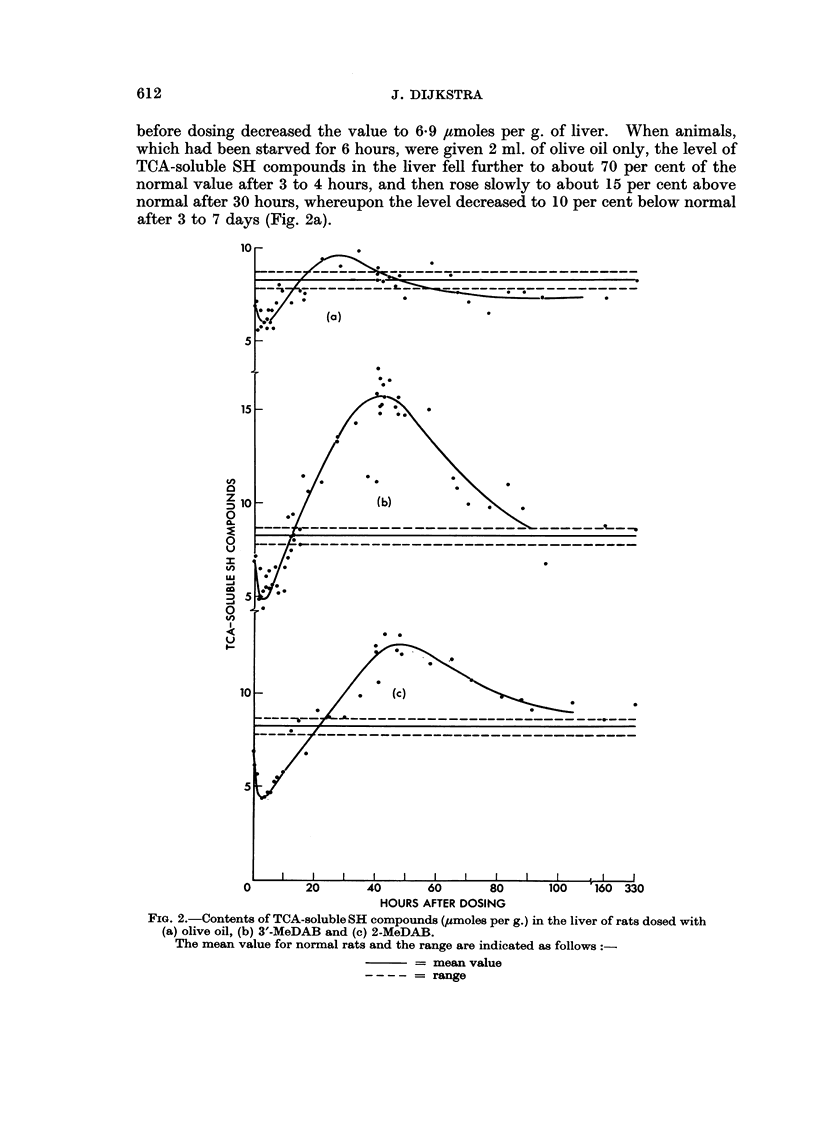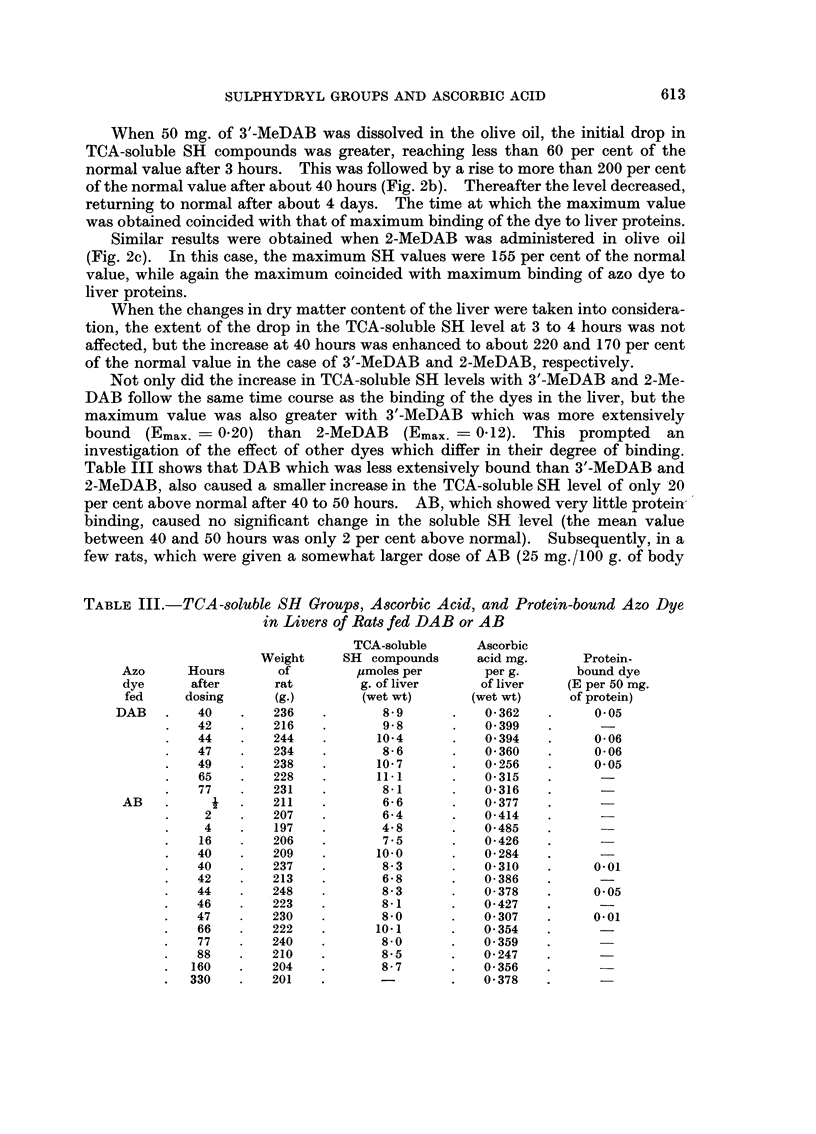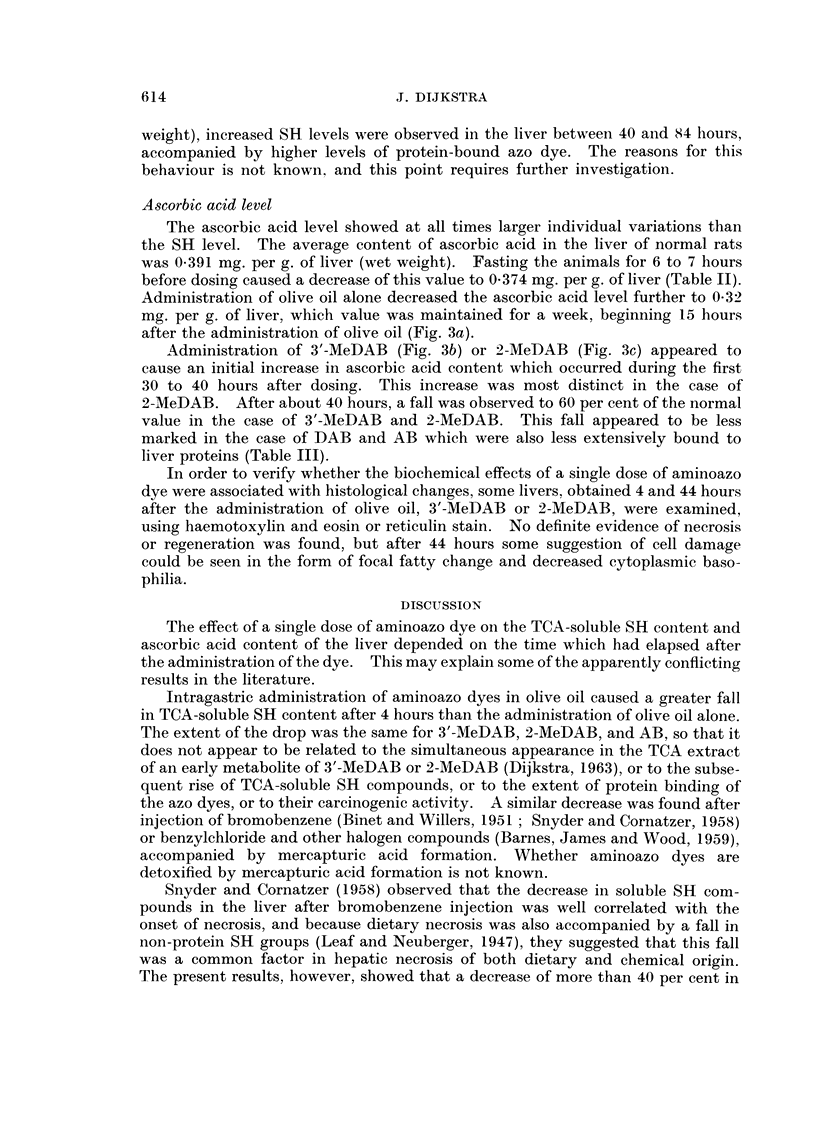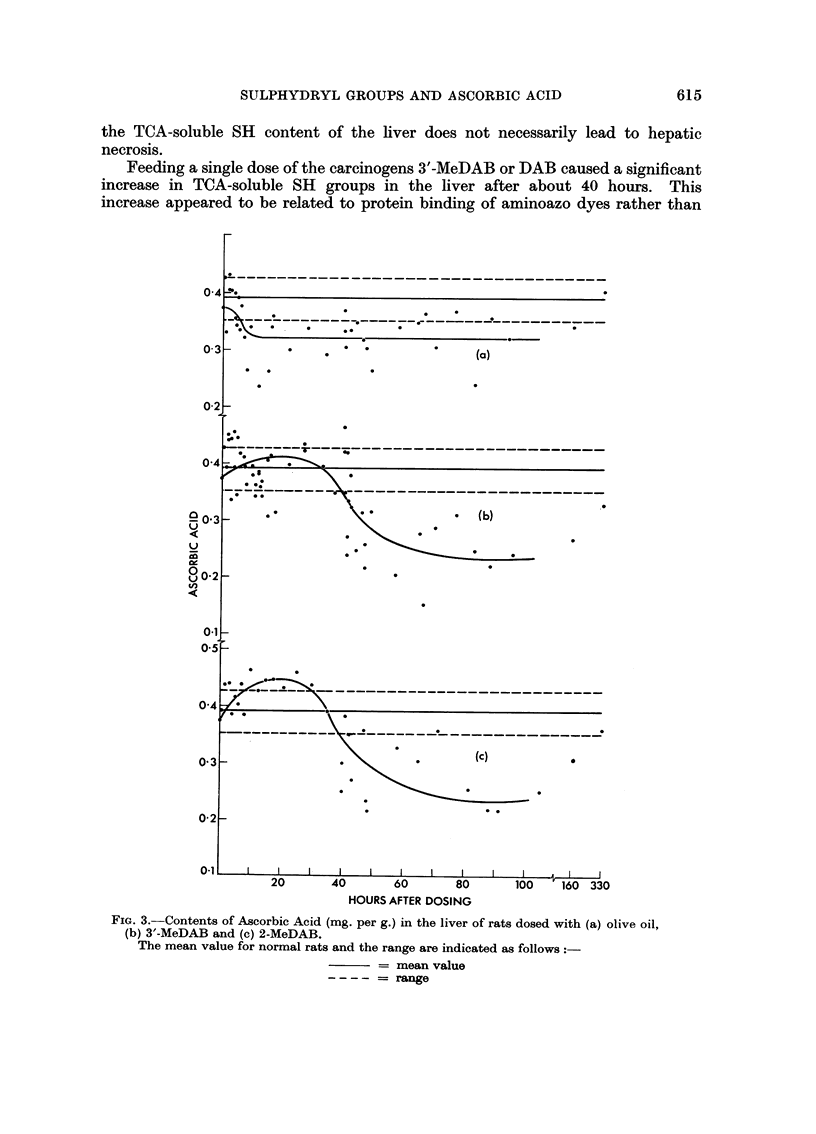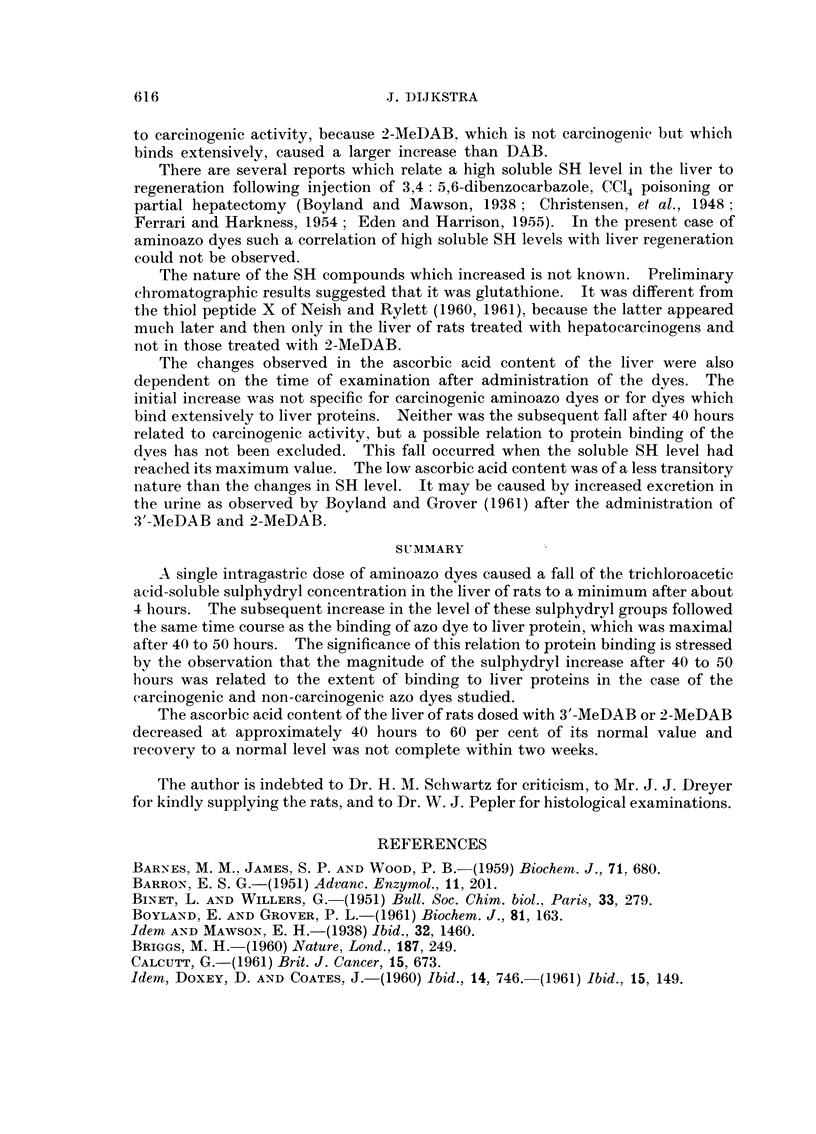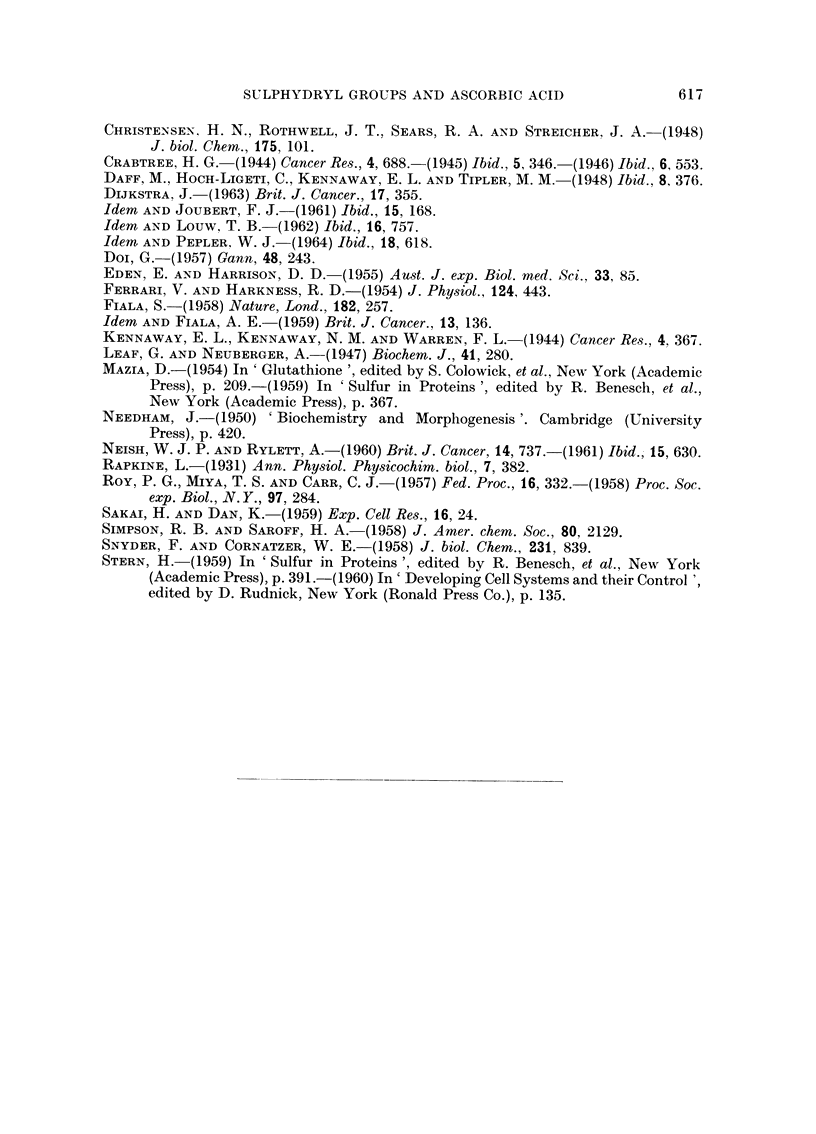# The Contents of Trichloroacetic Acid-soluble Sulphydryl Compounds and Ascorbic Acid in the Liver of Rats Fed Aminoazo Dyes: the Effect of a Single Large Dose of Dye

**DOI:** 10.1038/bjc.1964.70

**Published:** 1964-09

**Authors:** J. Dijkstra


### THE CONTENTS OF TRICHLOROACETIC ACID-SOLUBLE SULPHYDRYL COMPOUNDS AND ASCORBIC ACID IN THE LIVER OF RATS FED AMINOAZO DYES: THE EFFECT OF A SINGLE LARGE DOSE OF DYE

J. DIJKSTRA

*From the National Chemical Research Laboratory, South African Council for Scientific and Industrial Research, Pretoria, South Africa*

Received for publication March 16, 1964

FOLLOWING the original observation of Rapkine (1931) that the level of trichloroacetic acid (TCA)-soluble sulphydryl (SH) compounds in dividing sea urchin eggs fluctuated during cell division, considerable evidence has been accumulated to show that protein and non-protein SH groups play an important role in cell division (Needham, 1950; Barron, 1951; Mazia, 1954, 1959; Stern, 1959, 1960). Although it is not known how SH groups participate in this process, attempts have been made to relate carcinogenesis to a disturbance of this function. Thus Crabtree (1944, 1945, 1946) postulated that a primary carcinogenic disturbance is the binding of carcinogen to SH-containing cell constituents, and that anti-carcinogenic compounds which react with SH groups prevent this. On the other hand, Calcutt (1961) suggested that a rise in total SH level is essential for carcinogenesis and that anticarcinogenic substances inhibit this rise. Thus, the question of how SH groups take part in carcinogenesis has not been answered.

The question of whether it is the protein or non-protein SH groups which play a role also remains open. This is not surprising since there is a lack of agreement by different workers on the effects of carcinogens on SH levels. In particular, apparently conflicting effects of hepatocarcinogens on the non-protein SH content of the liver have been reported. Among the workers who gave a single dose of carcinogen, Boyland and Mawson (1938) noted a rise in the TCA-soluble SH level in the liver of mice for more than 20 days following an intraperitoneal injection of 3,4 : 5,6-dibenzocarbazole; Kennaway, Kennaway and Warren (1944) found that intraperitoneal injection of dimethylaminoazobenzene (DAB) into mice did not affect the metaphosphoric acid-soluble SH level; Roy, Miya and Carr (1958) observed that 5 hours after oral administration of DAB or $\beta$-naphthylamine to rats the sulphosalicylic acid-soluble SH level in the liver was lower than in the controls; while Neish and Rylett (1960, 1961) reported the appearance of an acidic thiol peptide in the liver of rats after an intraperitoneal injection of hepatocarcinogens, in contrast to non-carcinogenic compounds. In the case of continuous feeding of carcinogenic aminoazo dyes to rats, Roy, Miya and Carr (1957, 1958) reported slightly increased sulphosalicylic acid-soluble SH concentrations in the liver after 4 and 8 weeks followed by a depletion after 12 weeks if DAB was incorporated in a stock diet, and no increase if a low-protein diet was used; Fiala (1958) and Fiala and Fiala (1959) concluded that feeding 3′-methyl-DAB caused a late decrease of liver glutathione, but an increase of sulphosalicylic acid-soluble non-protein SH; and finally Calcutt, Doxey and Coates (1960) noted a slight increase of both metaphosphoric acid-soluble SH and total SH groups after feeding DAB, but in a later paper (1961) this effect was considered to be due to dietary factors rather than to the azo dye.

To tell which effects of carcinogens are significant for carcinogenesis and which are irrelevant, it is of value to compare the response to carcinogens with the response to closely related compounds which are non-carcinogenic. Of the above workers, only Neish and Rylett (1960, 1961) did this. It was therefore considered desirable, as part of a study of the metabolism and biochemical effects of carcinogenic aminoazo dyes, to follow the changes in TCA-soluble SH content of the liver after administration of both carcinogenic and non-carcinogenic members of this group.

Because changes in the soluble SH level in many conditions are paralleled by changes in the amount of ascorbic acid, the content of this acid in the liver was also determined. Workers who gave a single dose of hepatocarcinogen to mice found that the effect on the ascorbic acid content of the liver was not significant (Boyland and Mawson, 1938 ; Kennaway, Kennaway, and Warren, 1944). After feeding DAB continuously to rats, increased levels of ascorbic acid have been observed in the liver as well as in the hepatomas (Daff, *et al.*, 1948 ; Doi, 1957 ; Briggs, 1960).

This paper is concerned with changes following single intragastric doses of aminoazo dyes. The effects of continuous feeding will be reported in a subsequent paper (Dijkstra and Pepler, 1964).

### MATERIALS AND METHODS

*Reagents.*—The aminoazo dyes, 3′-methyl-4-dimethylaminoazobenzene (3′-MeDAB), 2-methyl-4-dimethylaminoazobenzene (2-MeDAB), 4-dimethylaminoazobenzene (DAB), and aminoazobenzene (AB), were prepared as described by Dijkstra and Louw (1962). They were dissolved in olive oil of BP quality. All other reagents were of analytical grade.

*Treatment of animals.*—Male albino rats (weight 220–260 g., unless otherwise stated) were given 50 mg. of dye in 2 ml. of olive oil by stomach tube between 9.00 and 10.00 p.m. and treated as described by Dijkstra (1963).

*Determination of dry matter.*—The dry matter content of the liver was determined by drying 1·0–1·6 g. portions of the left lateral lobe at 2° C. over $P_2O_5$ *in vacuo* to constant weight.

*Preparation of TCA extracts.*—The liver was extracted with TCA (2 ml. of 10 per cent TCA per g. of liver) as described by Dijkstra (1963). In order to calculate SH and ascorbic acid contents per g. of liver, the volume of extract obtained from 1 g. of liver was taken as 2·7 ml. This figure was based on the observation that the average dry weight of the liver was 30 per cent (range 27·3–32·7) for dye-treated rats.

*Ascorbic acid estimation.*—The method of Sakai and Dan (1959) was used. TCA extract (1·0 ml.) was added to 8 ml. of 2 per cent $HPO_3$, and the ascorbic acid was titrated in the cold with freshly prepared 2,6-dichlorophenol-indophenol (10 mg. per cent).

*Sulphydryl estimations.*—After titration of ascorbic acid, 1 ml. of 5 per cent KI and 2 drops of 1 per cent soluble starch were added to the slightly pink solution, and the SH compounds in the TCA extract were titrated with $5 \times 10^{-4}$ M $KIO_3$

In certain cases the SH groups were also determined by titration with MeHgI according to Simpson and Saroff (1958). TCA extract (1·0 ml.) was neutralised to pH 7·3 with 1 M $K_2HPO_4$ ; 5–10 ml. of $10^{-3}$ M MeHgI in toluene was added immediately and the mixture was stirred for 1 hour in the cold. After reaction, 0·2 ml. of the toluene layer was added to a mixture of 0·5 ml. pyridine-acetic acid (1 : 1) and 1 drop of 0·1 M ethylenediamine-tetraacetate, and the excess of MeHgI was titrated with 0·2 ml. dithizone in chloroform. The end-point of the titration was not sharp and the method was less precise than the iodometric method.

The recovery of reduced glutathione (Schwarz) was 99 per cent by the iodometric and 98 per cent by the MeHgI method.

Since the iodometric titration is not specific for SH groups, the observed increase in SH was checked with the MeHgI method in some TCA extracts. Table I shows that the two methods give identical results.

TABLE I.—*Comparison of the Values Obtained for TCA-soluble Sulphydryl Compounds in Rat Livers by the Iodometric and MeHgI Methods*

(μmoles SH per g. of liver)

| Iodometric method | MeHgI method |
|---|---|
| 6·9* | 6·8* |
| 12·2 | 12 |
| 13·5 | 13 |
| 15·2 | 15 |
| 15·6 | 15 |
| 16·3 | 16 |
| 16·6 | 16 |

* Liver from normal rat ; all other values for livers from rats given 3′-MeDAB.

*Stability of TCA extracts.*—Storage of the TCA extracts at 0° C. for 3 days caused a loss of 4–8 per cent of ascorbic acid and a decrease of 2–12 per cent in SH groups ; but over a period of 5 hours no noticeable changes could be detected if the extracts were kept under an atmosphere of nitrogen. The extracts were, therefore, kept under nitrogen and were usually titrated within one hour.

*Estimation of dye.*—The residues from a single TCA extraction were extracted with peroxide-free ether at room temperature to remove the TCA. In later experiments ethyl alcohol was used for this purpose. Alcohol-extractable dyes were then removed and the protein-bound azo dye determined as described (Dijkstra and Joubert, 1961). The amount of dye found per 50 mg. of protein was expressed in optical density units (E) as defined by Dijkstra and Louw (1962).

### RESULTS

The time and extent of maximum binding of the dyes to liver proteins in the rats used for the present experiments were similar to those described by Dijkstra and Louw (1962). The average dye levels ($E_{max.}$) at the time of maximum binding (40 to 50 hours after dosing) were 0·20, 0·12, 0·06, and 0·02 in the case of 3′-MeDAB, 2-MeDAB, DAB, and AB, respectively.

In the early experiments, the dry matter content of the livers was not determined but, following the report of Neish and Rylett (1960) that the dry matter content of rat liver was lower on the third and the sixth day after an intraperitoneal injection of carcinogenic compounds in contrast to non-carcinogens, in later experiments we determined the dry matter content of the liver of rats dosed with 3′-MeDAB or 2-MeDAB. The results are shown in Fig. 1. The dry matter content of the liver of our rats was generally higher than the values reported by Neish and Rylett (1960). Moreover, the dry matter content of the liver decreased in both the animals fed the carcinogen 3′-MeDAB and the non-carcinogen 2-MeDAB,

FIG. 1.—Dry matter content (percentage of wet weight) of the liver of rats dosed with 3′-MeDAB or 2-MeDAB.

reaching a minimum between 40 to 50 hours after dosing, when maximum binding of the dye to liver proteins occurred. Thereafter the values rose to levels which, in agreement with the findings of Neish and Rylett, were lower in the case of the carcinogen than in the case of the non-carcinogen.

*SH levels*

The content of TCA-soluble SH compounds in the liver of normal rats was 8·2 μmoles per g. of liver (wet weight) (Table II). Fasting the rats for 6 to 7 hours

TABLE II.—*Contents of TCA-soluble SH Compounds and Ascorbic Acid in the Liver of Normal Rats and of Rats after 7 Hours Fasting*

| | Normal rats | Fasted rats |
|---|---|---|
| Number of animals | 11 | 5 |
| Weight (g.) | 180–265 | 213–237 |
| SH compounds (μmoles per g. of liver, wet weight) | | |
| Range | 7·8–8·7 | 6·6–7·2 |
| Mean | 8·2 | 6·9 |
| Standard deviation | 0·3 | 0·3 |
| Ascorbic acid (mg. per g. liver, wet weight) | | |
| Range | 0·352–0·427 | 0·356–0·406 |
| Mean | 0·391 | 0·374 |
| Standard deviation | 0·028 | 0·019 |

before dosing decreased the value to 6·9 μmoles per g. of liver. When animals, which had been starved for 6 hours, were given 2 ml. of olive oil only, the level of TCA-soluble SH compounds in the liver fell further to about 70 per cent of the normal value after 3 to 4 hours, and then rose slowly to about 15 per cent above normal after 30 hours, whereupon the level decreased to 10 per cent below normal after 3 to 7 days (Fig. 2a).

Fig. 2.—Contents of TCA-soluble SH compounds (μmoles per g.) in the liver of rats dosed with (a) olive oil, (b) 3′-MeDAB and (c) 2-MeDAB.

The mean value for normal rats and the range are indicated as follows :—

——— = mean value
- - - - = range

When 50 mg. of 3′-MeDAB was dissolved in the olive oil, the initial drop in TCA-soluble SH compounds was greater, reaching less than 60 per cent of the normal value after 3 hours. This was followed by a rise to more than 200 per cent of the normal value after about 40 hours (Fig. 2b). Thereafter the level decreased, returning to normal after about 4 days. The time at which the maximum value was obtained coincided with that of maximum binding of the dye to liver proteins.

Similar results were obtained when 2-MeDAB was administered in olive oil (Fig. 2c). In this case, the maximum SH values were 155 per cent of the normal value, while again the maximum coincided with maximum binding of azo dye to liver proteins.

When the changes in dry matter content of the liver were taken into consideration, the extent of the drop in the TCA-soluble SH level at 3 to 4 hours was not affected, but the increase at 40 hours was enhanced to about 220 and 170 per cent of the normal value in the case of 3′-MeDAB and 2-MeDAB, respectively.

Not only did the increase in TCA-soluble SH levels with 3′-MeDAB and 2-MeDAB follow the same time course as the binding of the dyes in the liver, but the maximum value was also greater with 3′-MeDAB which was more extensively bound ($E_{max.} = 0{\cdot}20$) than 2-MeDAB ($E_{max.} = 0{\cdot}12$). This prompted an investigation of the effect of other dyes which differ in their degree of binding. Table III shows that DAB which was less extensively bound than 3′-MeDAB and 2-MeDAB, also caused a smaller increase in the TCA-soluble SH level of only 20 per cent above normal after 40 to 50 hours. AB, which showed very little protein binding, caused no significant change in the soluble SH level (the mean value between 40 and 50 hours was only 2 per cent above normal). Subsequently, in a few rats, which were given a somewhat larger dose of AB (25 mg./100 g. of body

TABLE III.—*TCA-soluble SH Groups, Ascorbic Acid, and Protein-bound Azo Dye in Livers of Rats fed DAB or AB*

| Azo dye fed | Hours after dosing | Weight of rat (g.) | TCA-soluble SH compounds µmoles per g. of liver (wet wt) | Ascorbic acid mg. per g. of liver (wet wt) | Protein-bound dye (E per 50 mg. of protein) |
|---|---|---|---|---|---|
| DAB | 40 | 236 | 8·9 | 0·362 | 0·05 |
| | 42 | 216 | 9·8 | 0·399 | — |
| | 44 | 244 | 10·4 | 0·394 | 0·06 |
| | 47 | 234 | 8·6 | 0·360 | 0·06 |
| | 49 | 238 | 10·7 | 0·256 | 0·05 |
| | 65 | 228 | 11·1 | 0·315 | — |
| | 77 | 231 | 8·1 | 0·316 | — |
| AB | ½ | 211 | 6·6 | 0·377 | — |
| | 2 | 207 | 6·4 | 0·414 | — |
| | 4 | 197 | 4·8 | 0·485 | — |
| | 16 | 206 | 7·5 | 0·426 | — |
| | 40 | 209 | 10·0 | 0·284 | — |
| | 40 | 237 | 8·3 | 0·310 | 0·01 |
| | 42 | 213 | 6·8 | 0·386 | — |
| | 44 | 248 | 8·3 | 0·378 | 0·05 |
| | 46 | 223 | 8·1 | 0·427 | — |
| | 47 | 230 | 8·0 | 0·307 | 0·01 |
| | 66 | 222 | 10·1 | 0·354 | — |
| | 77 | 240 | 8·0 | 0·359 | — |
| | 88 | 210 | 8·5 | 0·247 | — |
| | 160 | 204 | 8·7 | 0·356 | — |
| | 330 | 201 | — | 0·378 | — |

weight), increased SH levels were observed in the liver between 40 and 84 hours, accompanied by higher levels of protein-bound azo dye. The reasons for this behaviour is not known, and this point requires further investigation.

*Ascorbic acid level*

The ascorbic acid level showed at all times larger individual variations than the SH level. The average content of ascorbic acid in the liver of normal rats was 0·391 mg. per g. of liver (wet weight). Fasting the animals for 6 to 7 hours before dosing caused a decrease of this value to 0·374 mg. per g. of liver (Table II). Administration of olive oil alone decreased the ascorbic acid level further to 0·32 mg. per g. of liver, which value was maintained for a week, beginning 15 hours after the administration of olive oil (Fig. 3*a*).

Administration of 3′-MeDAB (Fig. 3*b*) or 2-MeDAB (Fig. 3*c*) appeared to cause an initial increase in ascorbic acid content which occurred during the first 30 to 40 hours after dosing. This increase was most distinct in the case of 2-MeDAB. After about 40 hours, a fall was observed to 60 per cent of the normal value in the case of 3′-MeDAB and 2-MeDAB. This fall appeared to be less marked in the case of DAB and AB which were also less extensively bound to liver proteins (Table III).

In order to verify whether the biochemical effects of a single dose of aminoazo dye were associated with histological changes, some livers, obtained 4 and 44 hours after the administration of olive oil, 3′-MeDAB or 2-MeDAB, were examined, using haemotoxylin and eosin or reticulin stain. No definite evidence of necrosis or regeneration was found, but after 44 hours some suggestion of cell damage could be seen in the form of focal fatty change and decreased cytoplasmic basophilia.

### DISCUSSION

The effect of a single dose of aminoazo dye on the TCA-soluble SH content and ascorbic acid content of the liver depended on the time which had elapsed after the administration of the dye. This may explain some of the apparently conflicting results in the literature.

Intragastric administration of aminoazo dyes in olive oil caused a greater fall in TCA-soluble SH content after 4 hours than the administration of olive oil alone. The extent of the drop was the same for 3′-MeDAB, 2-MeDAB, and AB, so that it does not appear to be related to the simultaneous appearance in the TCA extract of an early metabolite of 3′-MeDAB or 2-MeDAB (Dijkstra, 1963), or to the subsequent rise of TCA-soluble SH compounds, or to the extent of protein binding of the azo dyes, or to their carcinogenic activity. A similar decrease was found after injection of bromobenzene (Binet and Willers, 1951 ; Snyder and Cornatzer, 1958) or benzylchloride and other halogen compounds (Barnes, James and Wood, 1959), accompanied by mercapturic acid formation. Whether aminoazo dyes are detoxified by mercapturic acid formation is not known.

Snyder and Cornatzer (1958) observed that the decrease in soluble SH compounds in the liver after bromobenzene injection was well correlated with the onset of necrosis, and because dietary necrosis was also accompanied by a fall in non-protein SH groups (Leaf and Neuberger, 1947), they suggested that this fall was a common factor in hepatic necrosis of both dietary and chemical origin. The present results, however, showed that a decrease of more than 40 per cent in the TCA-soluble SH content of the liver does not necessarily lead to hepatic necrosis.

Feeding a single dose of the carcinogens 3′-MeDAB or DAB caused a significant increase in TCA-soluble SH groups in the liver after about 40 hours. This increase appeared to be related to protein binding of aminoazo dyes rather than

Fig. 3.—Contents of Ascorbic Acid (mg. per g.) in the liver of rats dosed with (a) olive oil, (b) 3′-MeDAB and (c) 2-MeDAB.

The mean value for normal rats and the range are indicated as follows :—

——— = mean value

- - - - = range to carcinogenic activity, because 2-MeDAB, which is not carcinogenic but which binds extensively, caused a larger increase than DAB.

There are several reports which relate a high soluble SH level in the liver to regeneration following injection of 3,4 : 5,6-dibenzocarbazole, $CCl_4$ poisoning or partial hepatectomy (Boyland and Mawson, 1938 ; Christensen, *et al.*, 1948 ; Ferrari and Harkness, 1954 ; Eden and Harrison, 1955). In the present case of aminoazo dyes such a correlation of high soluble SH levels with liver regeneration could not be observed.

The nature of the SH compounds which increased is not known. Preliminary chromatographic results suggested that it was glutathione. It was different from the thiol peptide X of Neish and Rylett (1960, 1961), because the latter appeared much later and then only in the liver of rats treated with hepatocarcinogens and not in those treated with 2-MeDAB.

The changes observed in the ascorbic acid content of the liver were also dependent on the time of examination after administration of the dyes. The initial increase was not specific for carcinogenic aminoazo dyes or for dyes which bind extensively to liver proteins. Neither was the subsequent fall after 40 hours related to carcinogenic activity, but a possible relation to protein binding of the dyes has not been excluded. This fall occurred when the soluble SH level had reached its maximum value. The low ascorbic acid content was of a less transitory nature than the changes in SH level. It may be caused by increased excretion in the urine as observed by Boyland and Grover (1961) after the administration of 3′-MeDAB and 2-MeDAB.

### SUMMARY

A single intragastric dose of aminoazo dyes caused a fall of the trichloroacetic acid-soluble sulphydryl concentration in the liver of rats to a minimum after about 4 hours. The subsequent increase in the level of these sulphydryl groups followed the same time course as the binding of azo dye to liver protein, which was maximal after 40 to 50 hours. The significance of this relation to protein binding is stressed by the observation that the magnitude of the sulphydryl increase after 40 to 50 hours was related to the extent of binding to liver proteins in the case of the carcinogenic and non-carcinogenic azo dyes studied.

The ascorbic acid content of the liver of rats dosed with 3′-MeDAB or 2-MeDAB decreased at approximately 40 hours to 60 per cent of its normal value and recovery to a normal level was not complete within two weeks.

The author is indebted to Dr. H. M. Schwartz for criticism, to Mr. J. J. Dreyer for kindly supplying the rats, and to Dr. W. J. Pepler for histological examinations.

### REFERENCES

BARNES, M. M., JAMES, S. P. AND WOOD, P. B.—(1959) *Biochem. J.*, **71**, 680.
BARRON, E. S. G.—(1951) *Advanc. Enzymol.*, **11**, 201.
BINET, L. AND WILLERS, G.—(1951) *Bull. Soc. Chim. biol., Paris*, **33**, 279.
BOYLAND, E. AND GROVER, P. L.—(1961) *Biochem. J.*, **81**, 163.
*Idem* AND MAWSON, E. H.—(1938) *Ibid.*, **32**, 1460.
BRIGGS, M. H.—(1960) *Nature, Lond.*, **187**, 249.
CALCUTT, G.—(1961) *Brit. J. Cancer*, **15**, 673.
*Idem*, DOXEY, D. AND COATES, J.—(1960) *Ibid.*, **14**, 746.—(1961) *Ibid.*, **15**, 149.

CHRISTENSEN, H. N., ROTHWELL, J. T., SEARS, R. A. AND STREICHER, J. A.—(1948) *J. biol. Chem.*, **175**, 101.
CRABTREE, H. G.—(1944) *Cancer Res.*, **4**, 688.—(1945) *Ibid.*, **5**, 346.—(1946) *Ibid.*, **6**, 553.
DAFF, M., HOCH-LIGETI, C., KENNAWAY, E. L. AND TIPLER, M. M.—(1948) *Ibid.*, **8**, 376.
DIJKSTRA, J.—(1963) *Brit. J. Cancer.*, **17**, 355.
*Idem* AND JOUBERT, F. J.—(1961) *Ibid.*, **15**, 168.
*Idem* AND LOUW, T. B.—(1962) *Ibid.*, **16**, 757.
*Idem* AND PEPLER, W. J.—(1964) *Ibid.*, **18**, 618.
DOI, G.—(1957) *Gann*, **48**, 243.
EDEN, E. AND HARRISON, D. D.—(1955) *Aust. J. exp. Biol. med. Sci.*, **33**, 85.
FERRARI, V. AND HARKNESS, R. D.—(1954) *J. Physiol.*, **124**, 443.
FIALA, S.—(1958) *Nature, Lond.*, **182**, 257.
*Idem* AND FIALA, A. E.—(1959) *Brit. J. Cancer.*, **13**, 136.
KENNAWAY, E. L., KENNAWAY, N. M. AND WARREN, F. L.—(1944) *Cancer Res.*, **4**, 367.
LEAF, G. AND NEUBERGER, A.—(1947) *Biochem. J.*, **41**, 280.
MAZIA, D.—(1954) In ' Glutathione ', edited by S. Colowick, *et al.*, New York (Academic Press), p. 209.—(1959) In ' Sulfur in Proteins ', edited by R. Benesch, *et al.*, New York (Academic Press), p. 367.
NEEDHAM, J.—(1950) ' Biochemistry and Morphogenesis '. Cambridge (University Press), p. 420.
NEISH, W. J. P. AND RYLETT, A.—(1960) *Brit. J. Cancer*, **14**, 737.—(1961) *Ibid.*, **15**, 630.
RAPKINE, L.—(1931) *Ann. Physiol. Physicochim. biol.*, **7**, 382.
ROY, P. G., MIYA, T. S. AND CARR, C. J.—(1957) *Fed. Proc.*, **16**, 332.—(1958) *Proc. Soc. exp. Biol., N.Y.*, **97**, 284.
SAKAI, H. AND DAN, K.—(1959) *Exp. Cell Res.*, **16**, 24.
SIMPSON, R. B. AND SAROFF, H. A.—(1958) *J. Amer. chem. Soc.*, **80**, 2129.
SNYDER, F. AND CORNATZER, W. E.—(1958) *J. biol. Chem.*, **231**, 839.
STERN, H.—(1959) In ' Sulfur in Proteins ', edited by R. Benesch, *et al.*, New York (Academic Press), p. 391.—(1960) In ' Developing Cell Systems and their Control ', edited by D. Rudnick, New York (Ronald Press Co.), p. 135.